# Emerging zoonotic diseases.

**DOI:** 10.3201/eid0707.017716

**Published:** 2001

**Authors:** G R Hansen, J Woodall, C Brown, N Jaax, T McNamara, A Ruiz

**Affiliations:** National Association of State Public Health Veterinarians, Topeka, Kansas, USA. GHansen@kdhe.state.ks.us


### Emerging Zoonotic Diseases

**Gail R. Hansen,* Jack Woodall,† Corrie Brown,‡ Nancy Jaax,§ Tracy McNamara,¶ and Alfonso Ruiz#**

*National Association of State Public Health Veterinarians, Topeka, Kansas, USA; †Federal University of Rio de Janeiro, Rio de Janeiro, Brazil, ‡University of Georgia, Athens, Georgia, USA; §USAMRIID, Frederick, Maryland, USA; ¶Wildlife Conservation Society, Bronx, New York, USA; and #Pan American Health Organization, El Paso, Texas, USA

### Emerging Zoonotic Bacterial and Parasitic Diseases

Corrie Brown from the University of Georgia discussed 15 diseases common to humans and animals, with a brief synopsis of how each disease is transmitted from animals to humans, the major animal reservoirs, and factors influencing the emergence of these diseases as human pathogens. The most important factors for emerging zoonotic diseases are (1) the transportation of humans and animals to new areas, (2) increased contact between animals and humans, (3) changes in the environment and husbandry practices, (4) a larger immunocompromised population, (5) increased recognition of diseases as zoonotic in origin, and (6) the discovery of new organisms not previously recognized. Domestic pets can transmit *Bartonella henselae*, *Sporothrix schenkii*, *Capnocytophaga carnimorsus*, *Echinococcus multilocularis* (alveolar hydatid disease), leishmaniasis, *Yersinia pestis* (plague), and ehrlichiosis. Emerging zoonotic disease agents transmitted by food animals include enteropathogenic *E. coli*, *Salmonella DT104* (which may have human origins), *Campylobacter* spp, and *Streptococcus iniae* (from farmed fish). The most common rat-transmitted disease in the United States is leptospirosis, which has a variety of serovars. Many of these diseases are difficult to diagnose and all of them can be fatal.

### Pathology of Animal Models for Filoviruses

Nancy Jaax, Colonel of the Veterinary Corps, USAMRIID, discussed Marburg and Ebola viruses, the only known filoviruses. Both Marburg and Ebola viruses have several serotypes, and all are African viruses, with the exception of Reston Ebola virus, which was traced to the Philippines. The natural histories, reservoirs, and epidemiologies of the viruses are largely unknown. In humans, persons with illness caused by filovirus infection usuall have influenzalike symptoms, with subsequent disseminated intravascular coagulopathy (DIC) and often generalized bleeding from body orifices. Pathologically, there is early and sustained infection of the mononuclear phagocyte system. There is no cure or vaccine, and treatment is symptomatic. The infections are transmitted by direct contact, and all body fluids contain large amounts of the rapidly replicating virus. Nonhuman primates have introduced the virus into human populations, but the animals appear to be amplifiers rather than reservoirs of the disease. The outbreaks associated with the filoviruses have been infrequent and temporally widely spaced, and few pathologic tissues are available for study. Only a few animal models are suitable for research, and research requires biosafety level (BSL) 4 containment. Guinea pigs and mice have been experimentally infected, but are poor predictors of primate response to treatment.

Address for correspondence: Gail R. Hansen, KDHE, 900 SW Jackson, Suite 1051, Topeka, KS 66612, USA; fax: 785-296-1127; e-mail: GHansen@kdhe.state.ks.us

### Exotic Birds as Sentinels for Human Disease

Tracy McNamara from the Wildlife Conservation Society discussed the West Nile virus outbreak that occurred in New York in the summer of 1999. More than 40 dead crows were recovered on the zoo grounds in the Bronx, New York. Necropsies ruled out bacteria, toxic pathogens, and several viral pathogens as the cause of death, but the exact cause remained elusive. Several North and South American birds died unexpectedly at the zoo in the following weeks. Abnormal pathologic changes were found in multiple organ systems in the birds, and encephalitis was only one of many manifestations of disease. The only common condition of the birds was that they were housed outdoors rather than indoors, but the petting zoo chickens and turkeys were unaffected. It was determined that a flavirus was causing the deaths, but further evaluation by the zoo was hampered by the lack of specific serologic tests for exotic animals and lack of adequate containment facilities. Emus are exquisitely susceptible to eastern equine encephalitis virus (an alphavirus), but the emus were healthy. From samples sent to USAMRIID, the birds were determined to have West Nile Virus. Birds are reservoirs for arboviruses but rarely die of them. In September 1999, several New York City residents were diagnosed with encephalitis caused by a flavivirus (originally thought to be St. Louis encephalitis virus but later determined to be West Nile Virus), and the connections were made. Zoo pathologists routinely take extensive tissue sections and blood samples on necropsied zoo animals, and these samples have been archived for more than 30 years. This archive may be valuable for future animal and human research.

### Plague in the Americas

Alfonso Ruiz from the Pan American Health Organization discussed plague, which is caused by *Yersinia pestis* and has caused three major pandemics in the history of civilization. Please see "Plague in the Americas" on page 539 for a more detailed article about this portion of the panel discussion.

